# Plantar Biomechanic Characteristics After High-Intensity Exercise in Young Runners With High-Arched Feet

**DOI:** 10.1155/abb/7834542

**Published:** 2025-05-20

**Authors:** Xindong Tao, Weiyi Lao, Yaoyao Zhong, Jihui Wang, Wei Ouyang

**Affiliations:** College of Physical Education and Health Sciences, Zhejiang Normal University, Jinhua 321004, China

**Keywords:** fatigue, high-arched feet, high-intensity ergometer exercise, plantar biomechanic characteristics

## Abstract

**Objective:** This study aimed to evaluate the plantar biomechanics in high-arched (HA) young runners after they performed a high-intensity (HI) ergometer exercise.

**Methods:** Eighteen collegiate runners with HA feet (age = 19.9 ± 0.6 years, height = 179.4 ± 3.5 cm, weight = 69.3 ± 4.9 kg, arch height index [AHI] = 0.43 ± 0.04) were tested. The participants performed a 5-min HI ergometer exercise. Besides measuring heart rate (HR), blood pressure (BP), and ratings of perceived exertion (RPE), the plantar biomechanic features were assessed before and after exercise.

**Results:** Postexercise, the participants exhibited a fatigue index (FI) of 87.6% ± 7.9%. The mean HR (HR_mean_) corresponded to 81.1% ± 5.4% of maximum HR (HR_max_). Notably, there was a significant drop (*p* < 0.001) in systolic BP (SBP) and diastolic BP (DBP) at the 10-min postexercise. The average RPE index was 17.3 ± 1.3. Specifically, the contact area of the hallux (T1) significantly increased (*p* < 0.05), while the contact area of toes 2-5 (T2-5) and the 5th metatarsal (M5) significantly decreased (*p* < 0.05). Plantar pressure significantly increased in T1 (*p* < 0.05), but significantly decreased in M5 and T2-5 (*p* < 0.05). The force–time integral (FTI) in the forefoot and vertical ground reaction force (VGRF) during heel fully struck and forefoot push off elevated, while the foot progression angle (FPA) significantly increased (*p* < 0.01).

**Conclusions:** Our findings indicate that HI ergometer exercise has significant impacts on the biomechanic features of HA young runners. Specifically, we observed modifications in plantar area and pressure, FTI, and VGRF, especially in the medial arch and combined with outward rotation of the feet. These results can offer insights to inform future investigations on gait training interventions aimed at reducing the risk of lower extremity injuries in HA young runners.

## 1. Introduction

Running is a widely embraced exercise known for its health benefits [1, 2]. However, running-related injuries are prevalent, with incidence rates ranging from 4.26 to 33.07 per 1000 h of exposure, depending on the runner's experience [2]. These injuries can significantly impact health, quality of life, and productivity, underscoring the need for targeted prevention strategies. Among the factors influencing injury risk, foot arch structure plays a critical role due to its distinct biomechanical profile [2].

High-arched (HA) feet, defined by an elevated longitudinal arch, have become increasingly common among adolescents over the past two decades [3–5]. This foot structure is associated with unique biomechanical characteristics, including higher ground reaction forces (GRFs), greater stiffness in the lower extremities, and an increased vulnerability to overuse injuries such as ankle instability and sprains, plantar fasciopathy, metatarsalgia, and stress fractures [6–9]. Interestingly, HA feet may also offer advantages, such as improved running speed and stronger plantar muscles [10, 11]. Given these dual aspects, understanding these nuances is essential for tailoring training and rehabilitation programs to HA runners, yet the acute effects of high-intensity (HI) exercise on their plantar biomechanics remain underexplored.

Fatigue from HI exercise modifies lower extremity biomechanics and plantar pressure patterns, increasing the risk of injury [12, 13]. Critical measures such as GRF and foot progression angle (FPA) are tied to both performance and injury potential [7, 14–16], and plantar pressure platforms are key tools for evaluating these shifts [17]. While prior research has investigated high-impact activities like running and jumping in HA individuals [9, 18, 19], findings often show increased lateral plantar pressure after treadmill running [9] or repeated jumps in badminton players [19]. However, these studies struggle to separate the effects of fatigue from those of impact forces, complicating efforts to pinpoint fatigue's specific role. Low-impact HI exercises, such as ergometer cycling, provide an opportunity to isolate fatigue effects without the interference of impact, yet this approach remains underexplored.

This study explores plantar biomechanical changes in young HA runners after a 5-min HI ergometer (cycling) protocol—a low-impact approach that isolates fatigue effects [20, 21]. Using the Footscan platform, we measured contact area, pressure, force–time integral (FTI), vertical GRF (VGRF), and FPA. Our study stands out due to the isolation of fatigue effects, comprehensive biomechanical analysis, and providing insights for gait training and injury prevention tailored to HA runners, a group prone to injury. This research deepens our knowledge of HA foot biomechanics during HI exercise, delivering practical value for sports science and clinical applications.

## 2. Materials and Methods

### 2.1. Participants

Eighteen young, healthy male runners were recruited from the Physical Education College of Zhejiang Normal University. Only male participants were selected to control for potential sex-based physiological differences that could influence the study outcomes, such as variations in foot structure, hormonal influences, and running biomechanics. Participants were screened for study exclusion criteria, which included contraindications to maximal exercise, smoking, current or family history of cardiovascular or cerebrovascular disease, and use of dietary supplements within 12 weeks before study enrollment. All participants underwent a series of normal physical exams, such as body mass index (BMI) and basal metabolic rate (BMR) were assessed using the InBody 570 body composition analyzer (Inbody USA, Cerritos, California, USA). To ensure all participants possess good body composition, muscular endurance, muscular strength, cardiovascular endurance, and flexibility, their fitness status was evaluated through a series of standardized tests (specified in Table 1). Additionally, participants' running proficiency was assessed based on their training history and competition experience to ensure a relatively homogeneous level of running ability among the sample, thereby minimizing potential variability in performance outcomes.

Arch height was measured using the arch height index (AHI), which is calculated as the dorsal height at 50% of the total foot length divided by the truncated foot length (the bony distance from the most posterior point of the calcaneus to the most distal point of the first metatarsal head). The AHI is a reliable and valid method of assessing arch height [22]. Participants were classified based on their AHI: those with AHI ≥ 0.365 were categorized as having high arches, while those with AHI ≤ 0.275 were classified as having low arches [22]. The study protocol was approved by the Human Research Ethics Committee (ZSRT2021071), Zhejiang Normal University. All participants provided written informed consent before taking part in the study, and all methods were performed in accordance with relevant guidelines and regulations.

### 2.2. HI Ergometer Exercise Procedure

Monark ergometer exercise can be used to evaluate the maximal work, anaerobic ability, and power drop of the athlete's lower limbs [23]. In this study, all participants reported once to our laboratory to undergo testing procedures. The participant sat on the cycle ergometer (Monark 894E, Varberg, Sweden) with an upright position of the torso and then carried out a 3 min warm-up activity with 1 kg resistance to make the heart rate (HR) reach 70% maximum HR (HR_max_). At the end of the warm-up session, the load (6.5% kg/kg) is applied immediately; the participant continues to exercise for 5 min, followed by 2 min cool-down cycling without load. The constant load protocol was modified based on a recent review [24], two previous reports [25, 26], and the Monark 894 manual. Constant load ergometer test performed at a work rate below the peak work rate was shown to be more convenient to verify a maximal effort and VO_2max_ [27]. Power drop is also called fatigue index (FI) [23]. FI(%)=100 × ([Power_max_ − Power_mini_]/Power_max_). The smaller value of FI indicates the stronger athlete's antifatigue ability and vice versa. Ratings of perceived exertion (RPE) strongly correlate with HR during ergometer cycling exercise [28]. Therefore, the RPE using a 6–20 Borg scale [29] was recorded immediately after the ergometer exercise. Additionally, the HR was monitored during the whole exercise process to help verify the exercise intensity.

### 2.3. HR and Blood Pressure (BP) Measurements

Since differences in genetic factors profoundly shape the physical parameters (i.e., height, weight, and BMI) of Asians, Europeans, and/or Americans [30], we adopted a newly published HR_max_ estimation formula designed for young male Asians (15–24 years, HR_max_ = 214–0.8 × age) [31] to predict the HR_max_ in our participants. Actual HR was measured using a Firstbeat monitor (Firstbeat Sports team, Firstbeat Technologies Ltd., Jyväskylä, Finland). Systolic BP (SBP)/diastolic BP (DBP) was measured with an automatic BP cuff (HEM-7121, Omron Healthcare, Lake Forest, IL, USA). The HR was measured twice at rest, 10 min before, during, and after the ergometer exercise. The percentage HR decline at 2 min of recovery was used to express the rate of HR recovery after exercise. The recovery rate at 2 min postexercise (%) = 100 × [(HR_max_ during exercise − HR at 2 min postexercise)/HR_max_ during exercise [32]. The right brachial arterial BP was measured twice at 10 min before and after the ergometer exercise in participants in the supine position. These measurements were repeated for all participants under the same experimental condition.

### 2.4. Plantar Biomechanical Assessment

All participants had HA and no history of gait abnormalities such as foot varus and foot valgus. Before and immediately after the participants performed the 5 min HI ergometer exercise, we used the FootScan 1.5 m pressure pad (RSscan International, Olen, Belgium, sampling rate, 300–400 Hz; number of sensors, 12,288/1.5 m, the sensor size, 5 × 7.6 mm, 4 sensors/cm^2^; the pressure range, 0.7–155 N/cm^2^, the accuracy of 3.3%) to test the plantar area and pressure. The pressure pad was placed in the middle of a 5 m long mat. Participants uniformly started walking on the right foot, with the right foot first outside the pressure pad and the left foot walking first on the pressure pad [33]. The participants walked to the end of the 5 m-long mat at a comfortable, natural pace.

**Figure 1:**
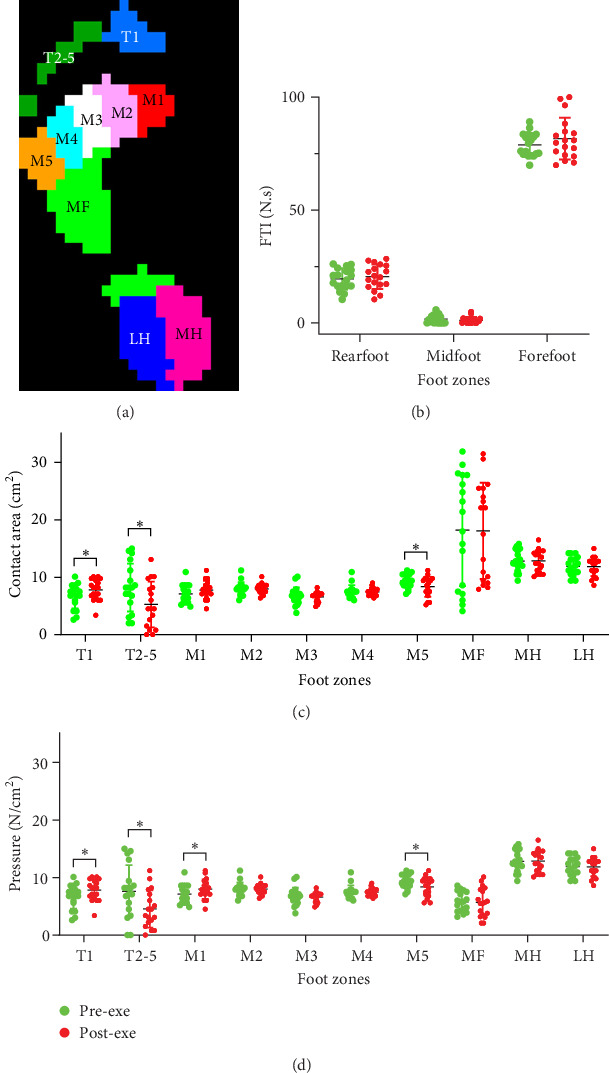
The alternations of the contact area and plantar pressure after participants performed the HI ergometer exercise. (A) A captured image shows the subdivision area of the left foot. The subdivisions are: T1, T2-5, M1, M2, M3, M4, M5, MF, MH, and LH. (B) The FTI changes in the forefoot, midfoot, and rearfoot pre- and post-HI ergometer exercise, respectively. (C) The effects of HI ergometer exercise on participants' foot contact areas. (D) The effects of HI ergometer exercise on participants' plantar pressure. The data in the figure is represented by mean ± SD, *n* = 18; *⁣*^*∗*^*p* < 0.05, paired *t*-test. T1, hallux; T2-5, toes 2–5; M1, first metatarsal; M2, second metatarsal; M3, third metatarsal; M4, fourth metatarsal; M5, fifth metatarsal. LH, lateral heel; MF, midfoot; MH, medial heel.

FootScan comes with software for assessing 10 plantar partitions (hallux, T1; toes 2–5, T2-5; first metatarsal, M1; second metatarsal, M2; third metatarsal, M3; fourth metatarsal, M4; fifth metatarsal, M5; midfoot, MF; medial heel, MH; lateral heel, LH (Figure 1A). Before data collection, all participants were given clear instructions regarding the testing protocols. In addition, they were instructed to wear loose-fitting, comfortable clothing that would not restrict lower limb motion. All participants were asked to complete 5 min of walking trials along the FootScan platform before dynamic data collection commenced. To increase the reliability of the collected data, each participant underwent three trials of the testing protocol [34, 35], and the contact area (cm^2^), plantar pressure (N/cm^2^), FTI (also defined as an impulse, N.s), VGRF (N) and FPA (foot axis angle, degree) were averaged across the trials for subsequent analysis. A trial was considered valid if it met the following criteria: (1) the presence of at least two complete footprints on the pressure platform, (2) the presence of a visible heel-strike pattern during walking, and (3) no noticeable adjustments or deviations in gait pattern when crossing the pressure platform [36].

To maintain data independence and reliability, we selected only the left foot of each participant for analysis [17, 18]. FTI is increasingly used to define the cumulative effect of force over time in a certain area of the foot and thus provides a value for the total load exposure of a foot sole area during one step [9, 37]. Accumulation of plantar force may lead to tissue damage, so this variable can also be used to assess exercise fatigue and injury risk [9]. FPA is defined as the angle made by the long axis of the foot from the heel to the 2nd metatarsal in the stance phase and the line of progression of gait. A negative FPA indicates that in-toeing means the foot rotates inward, and a positive FPA, out-toeing indicates the foot rotates outward [38]. Out-toeing foot posture increases the load borne by the medial column support structures (e.g., bones, ligaments, muscles), thereby increasing the risk of injury to these structures. Therefore, in addition to the assessment of contact area and plantar pressure, we also assessed the changes in FTI, VGRF, and FPA in this study. During the stance phase, all participants exhibited consistent timing in their heel strike and forefoot push before toe-off, with a range of 71.13 ms for heel strike and 162.03 ms for forefoot push. Therefore, we collected the values of the first (heel strike) and second (forefoot push) VGRF peaks from within these time ranges for further analysis. Given the narrow range of BMI (21.6 ± 1.4 kg/m^2^) and weight (69.3 ± 4.9 kg) among the participants, it is unlikely that changes in foot shape due to differences in body weight or load-bearing conditions would significantly impact the results [39, 40]. Therefore, the VGRF values were not normalized to the participant's weight. The outcomes of the plantar contact area, pressure, FTI, FPA, and the aforementioned HR and BP were evaluated by three independent blinded examiners.

### 2.5. Statistical Analyses

All data are expressed as mean ± standard deviation (mean ± SD) and were assessed the normal distribution using Kolmogorov and Smirnov test. To compare changes before and after exercise, paired *t*-tests were conducted on plantar contact area, plantar pressure, FTI, VGRF, and FPA. To assess the magnitude of these changes, effect sizes were calculated using Cohen's *d*, with the following interpretive thresholds: 0.2, small effect; 0.5, medium effect; 0.8, large effect. For the analysis of plantar contact area and pressure across four specific regions (T1, T2-5, M1, M5), a Bonferroni correction was applied to adjust for multiple comparisons. This adjustment set the significance threshold at *p* < 0.0125 (calculated as 0.05 divided by four regions). To evaluate the risk of overuse injury, a Pearson correlation analysis was performed to explore the relationship between the power drop value and the changed value of the contact area of the plantar T1, T2-5 area, the change of pressure, and the angle deviation of the foot axis. All statistical analyses were performed using Prism 9 (GraphPad, San Diego, CA, USA). *⁣*^*∗*^*p* < 0.05 and *⁣*^*∗∗*^*p* < 0.01 values for statistically significant differences.

## 3. Results

### 3.1. Participant Characteristics

The basic characteristics of the participants are assessed. The age of the participants is 19.9 ± 0.6 years old; height is 179.4 ± 3.5 cm, weight is 69.3 ± 4.9 kg, BMI is 21.6 ± 1.4 kg/m^2^, HR is 65.4 ± 6.5 beats per minute (BPM), SBP is 120.6 ± 8.2 mmHg, DBP is 65.6 ± 7 mmHg, body fat rate is 13.5% ± 3.6%, BMR is 7264.9 ± 391.2 kJ (Table S1). The data indicated that all participants were in good health. The fitness test results (Table 1) showed that all participants possessed good physical fitness, including static balance, lower and upper body strength, lower and upper body flexibility, agility/dynamic balance, walking speed, and aerobic capacity. The AHI of participants is 0.43 ± 0.04, confirming that all participants had HA feet [22].

### 3.2. HI Ergometer Exercise

**Figure 2:**
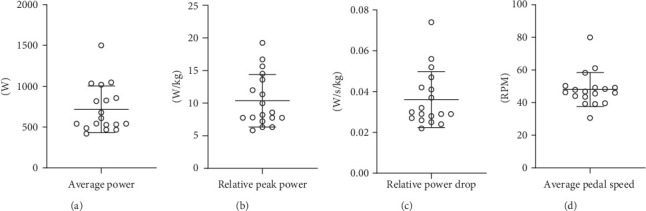
The performance of the participants during 5 min HI ergometer cycling. (A) Average output power (watt, W). (B) Relative peak power (W/Kg). (C) The value of the power drop compared to weight per second. (D) Average pedal speed (revolutions per minute, RPM). The data in the figure is represented by mean ± SD, *n* = 18.

After the participants performed a 5 min HI ergometer exercise, the average output power was 717.6 ± 278.5 W, and the relative peak power was 10.4 ± 3.9 w/kg. The relative power drop was 0.04 ± 0.01 w/s/Kg, and the average pedal speed was 48.1 ± 10.2 RPM (Figure 2). Moreover, the FI outcome was 87.6% ± 7.9%, indicating that all participants reached a fatigued state after ergometer cycling.

Based on ACSM guidelines, exercise intensity can be expressed as a percentage of a person's HR_max_ (high intensity, 76%–96% HR_max_) or as an index of RPE (high intensity, RPE index ≥ 5 and ≥15 in 10- and 20-point scales, respectively). In this study, the HR_max_ was 178.4 ± 6.9 BPM during the ergometer exercise, while mean HR (HR_mean_) was 160.6 ± 10.8 BPM, and equal to 81.1% ± 5.4% HR_max_ (Table 2). Therefore, the exercise intensity was characterized as high intensity [41]. The HR recovery rate at 2 min postexercise was 29.9% ± 4.7%. There is a significant drop in SBP (delta SBP = −6.2 ± 4.3 mmHg, *p* < 0.001) and DBP (delta DBP = −4.8 ± 3.7 mmHg, *p* < 0.001) compared to pre-exercise values (Table 2). RPE was recorded immediately after the exercise. Among the 18 participants, 2 rated the exercise as “hard," 11 as “very hard," and 5 as “extremely hard” (Table S2). Therefore, 16 out of 18 participants reported “very hard” or “extremely hard,” with an average index of RPE 17.3 ± 1.3.

### 3.3. Changes in Contact Area and Plantar Pressure

Data analysis revealed that changes in contact area and plantar pressure primarily occurred in the T1, T2-5, M1, and M5 regions after ergometer cycling (Figure 1A). The rearfoot area included the medial and lateral heels, the midfoot area included the arch, and the forefoot area included the phalanges and metatarsals. Following the ergometer exercise, forefoot (pre-exe: 78.9 ± 5.1 N.s; post-exe: 81.6 ± 9.0 N.s) and rearfoot (pre-exe: 19.4 ± 4.6 N.s; post-exe: 20.5 ± 5.3 N.s) FTI increased, while the midfoot (pre-exe: 1.8 ± 1.7 N.s; post-exe: 1.3 ± 1.2 N.s) FTI decreased. However, these changes were not statistically significant (Figure 1B).

After Bonferroni correction for multiple comparisons (adjusted *α* = 0.01), the T1 area significantly increased from 6.7 ± 0.01 to 7.8 ± 1.8 cm^2^ (*p* < 0.05, Cohen's *d* = 0.61), while the T2-5 area of significantly decreased from 8.2 ± 4.1 to 5.3 ± 3.9 cm^2^ (*p* < 0.05, Cohen's *d* = 0.73). The M1 area of increased from 7.1 ± 1.5 to 7.8 ± 1.5 cm^2^ (*p*=0.09, Cohen's *d* = 0.41), while the M5 area significantly decreased from 9.3 ± 1.2 to 8.3 ± 1.7 cm^2^ (*p* < 0.05, Cohen's *d* = 0.65; Figure 1C). Pearson correlation analysis showed that there is a positive correlation between the FI and increased T1 area (*r* = 0.557; *p*=0.016) or decreased T2-5 area (*r* = 0.51; *p*=0.031).

Similar trends were observed in plantar pressure changes. Plantar pressure in T1 increased significantly from 6.7 ± 2.0 to 7.8 ± 1.8 N/cm^2^ (*p* < 0.05, Cohen's *d* = 0.54), while T2-5 decreased from 7.6 ± 4.4 to 4.5 ± 3.1 N/cm^2^ (*p* < 0.05, Cohen's *d* = 0.73). M1 pressure increased from 7.1 ± 1.5 to 8.0 ± 1.6 N/cm^2^ (*p* < 0.05, Cohen's *d* = 0.52), and M5 decreased from 9.4 ± 1.2 to 8.4 ± 1.6 N/cm^2^ (*p* < 0.05, Cohen's *d* = 0.59) (Figure 1D). FI positively correlated with the increase in T1 pressure (*r* = 0.513, *p*=0.029) and the decrease in T2-5 pressure (*r* = 0.532, *p*=0.023). To adjust for multiple comparisons across the four regions (T1, T2-5, M1, M5), the Bonferroni correction was applied, setting the significance threshold at *p* < 0.0125 (0.05/4). These results showed the predominant contact area and plantar pressure changes in the forefoot zone. The contact area and plantar pressure increased in the forefoot medial subdivisions; in contrast, the area and pressure values decreased in the forefoot lateral subdivisions.

### 3.4. The GRF Changes Across the Foot During the Stance and Preswing Phases

**Figure 3:**
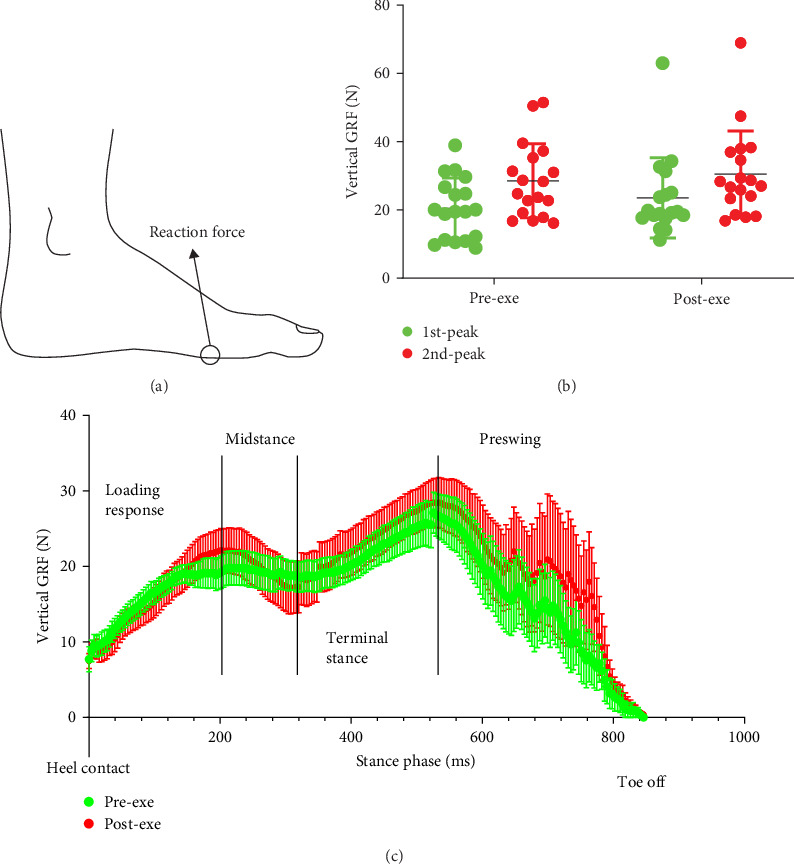
The alternations of VGRF peak values and real-time curves of VGRF during the stance and preswing phases after participants performed the HI ergometer exercise. (A) A schematic diagram shows the VGRF in the left foot. (B) The alternations of VGRF values during heel fully struck (1st peak) and forefoot push off (2nd peak) pre- and postexercise. (C) Real-time VGRF curves across the foot during stance and preswing phases pre- and postexercise. The data in the figure is represented by mean ± SD, *n* = 18. VGRF, vertical ground reaction force.

The FootScan pressure pad can effectively detect the GRF of the ground against the foot plantar when the participant is walking, running, or jumping.  Figure 3A presents an example of the VGRF recorded in this study. The VGRF provides an estimate of landing intensity and reflects the forces experienced during the stance phase. VGRF showed nonsignificant increases during heel strike (1st peak: pre: 20.56 ± 8.62 N; post: 23.33 ± 12.45 N, *p*=0.42, Cohen's *d* = 0.26) and forefoot push-off (2nd peak: pre: 28.56 ± 10.23 N; post: 30.00 ± 12.34 N, *p*=0.70, Cohen's *d* = 0.13, Figure 3B). While these changes did not reach statistical significance (all *p* > 0.05 after Bonferroni correction, adjusted *α* = 0.025), the effect sizes indicate trivial to small magnitudes of change. The real-time VGRF curve indicated greater variability during the preswing phase postexercise (Figure 3C). This finding has practical implications for athletes and clinicians, as it suggests that training programs should incorporate exercises aimed at strengthening the foot's intrinsic muscles and optimizing propulsion mechanics to minimize fatigue-related performance declines and injury risks.

### 3.5. The FPA Changes

**Figure 4:**
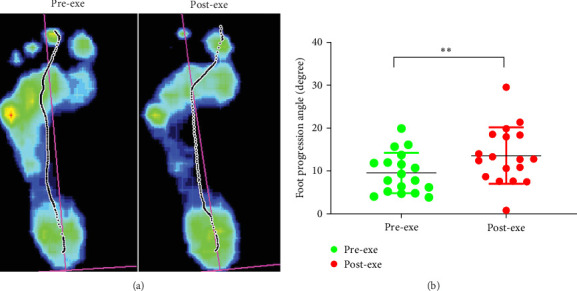
The FPA changes after participants perform HI ergometer exercises. (A) The screenshot of the representative experiment shows that the angle of the participant's left foot long axis (indicated by the red vertical line on the foot) changes before (A1) and after (A2) the participant performs the HI ergometer exercise. (B) Summary of the participant's left foot FPA changes. The data in the figure are represented by mean ± SD, *n* = 18; *⁣*^*∗∗*^*p* < 0.01, paired *t*-test. FPA, foot progression angle.

The FPA significantly increased from 9.5 ± 4.6 to 12.2 ± 7.7 degrees postexercise (*p* < 0.01, Cohen's *d* = 0.74, Figure 4), indicating increased foot external rotation. This shift suggests heightened stress on medial foot structures, including bones, ligaments, and muscles, particularly in HA individuals. Pearson correlation analysis revealed a positive correlation between postexercise FPA changes and FI (*r* = 0.531; *p*=0.023). From a clinical perspective, these findings underscore the importance of fatigue management in athletes, especially those with HA feet. Excessive FPA increases pronation-related stress, elevating the risk of overuse injuries such as plantar fasciitis and medial tibial stress syndrome. Strengthening medial foot structures and incorporating targeted gait training may help mitigate these risks. Clinicians and trainers should emphasize foot alignment monitoring during HI activities to optimize performance and reduce injury risk.

## 4. Discussion

The biomechanic characteristics of the foot, particularly the arch structure, play a critical role in sports involving HI activities, influencing performance optimization and injury prevention. The study investigated the effects of HI ergometer exercise on plantar contact area, pressure, VGRF, and FPA in young runners with HA feet. Exercise intensity was validated using HR, BP, and the RPE, consistent with ASCM guidelines [41] and prior research [29, 41]. Participants (~20 years old, BMI ~22) achieved a mean HR_max_ of ~178 BPM and an RPE of ~17, aligning with maximal effort studies [32, 42]. However, the HR recovery rate at 2 min postexercise was ~30%, lower than the ~35%–38% reported at 1 min in similar studies [32, 42], possibly due to a 2 min cool-down phase. Additionally, SBP/DBP decreased significantly 10 min postexercise (delta SBP: ~ −6 mmHg; delta DBP: ~5 mmHg), mirroring trends in maximal effort ergometer studies [32]. To assess lower extremity anaerobic capacity and fatigue, we set resistance at 6.5% of body weight for a 5-min HI ergometer exercise, adapted from shorter protocols using 8.5% [43] or 10%–11% [44] of body weight. This duration and intensity effectively induced fatigue while prioritizing participant safety. Given HA feet's association with specific injury risks, such as ankle injuries and overuse syndromes [7, 8], we exclusively studied HA participants for consistency.

Foot plantar pressure refers to the force acting between the foot and the ground during locomotion, which can be affected by the size of the test area [37]. Therefore, in this study, we examined changes in FTI in large areas of the plantar (rearfoot, midfoot, forefoot) before and after exercise. After the ergometer exercise, we observed clear upward trends in the FTI of the rearfoot and forefoot plantar regions. The results of this study partially agree with some previous findings [9, 18, 19]. In the plantar subregions, plantar contact area and pressure significantly increase at T1 and M1 but significantly decrease at T2-5 and M5. The results partially align with a previous report showing that people with HA feet had larger center of pressure excursions than people with normal or pronated feet [45]. Notably, our findings are comparable to those of a previous study, which reported increased T1, T2-5, M1, and M3 pressures but decreased M2, M4-5, and LH pressure after exhaustive treadmill running in HA individuals [9]. The discrepancy—particularly opposite trends in M4-5 and LH—likely arise from exercise modality differences. This medial shift in our study may reflect fatigue-related compensatory mechanisms, such as reduced muscle function and increased joint laxity, leading to greater pronation, medial arch flattening, and tibial internal rotation [11, 46]. These changes could elevate the long-term risk of overuse injuries, such as plantar fasciitis or medial tibial stress syndrome [8, 9]. Comparatively, badminton players with HA feet show increased forefoot and rearfoot contact areas (e.g., T1) and higher pressure at T1, T2-5, MH, and LH [19], driven by high-impact jumps and landings. In contrast, our low-impact cycling results highlight a distinct medial loading pattern, underscoring the influence of exercise type on plantar biomechanics.

VGRF increased during the heel strike and toe-off phases postexercise, suggesting altered gait dynamics. While prior studies report inconsistent VGRF changes postexercise [47, 48], our findings indicate that HI ergometer exercise may heighten impact forces in HA runners. This could stem from fatigue-induced reductions in shock absorption, potentially increasing risks of stress fractures or impact-related injuries [49, 50]. In this study, FPA increased significantly postexercise, reflecting an outward gait shift that correlated positively with participants' FI. This external rotation may increase knee valgus torque, elevating anterior cruciate ligament (ACL) injury risk [16, 51]. Persistent exercise in this fatigued state could further amplify this risk [52]. Our results align with studies reporting ankle inversion and elevated foot posture index (FPI) postexercise [18, 19], suggesting a compensatory mechanism that may overload medial foot structures. Unlike HA feet, flat-footed individuals often exhibit excessive pronation and medial loading at rest [45], which fatigue may exacerbate, potentially leading to medial shin splints or knee pain [7]. Normal-arched individuals, as seen in moderate running studies, show decreased T1 contact area and increased M1 pressure postexercise [18], differing from our HA findings. These variations suggest that HA feet shift medially under fatigue, while flat feet may intensify existing medial loads, highlighting the need for comparative studies across foot types.

Several limitations should be acknowledged. First, the use of standardized footwear during testing may have masked individual variations in foot biomechanics influenced by habitual shoe characteristics, such as arch support or cushioning properties [53, 54]. Second, muscle fatigue and activation patterns in the foot and lower leg, which could exacerbate plantar loading asymmetries [12, 47], were not directly measured. Future studies should incorporate electromyography to quantify fatigue-related neuromuscular adaptations. Third, the focus on young HA runners' limits generalizability to populations with different foot types (e.g., low-arched), ages, or athletic backgrounds [5, 55]. Additionally, the use of a cycling ergometer, while controlled, may not fully replicate running-specific biomechanical demands [20]. Longitudinal investigations are warranted to establish causal links between acute plantar loading shifts and overuse injuries like plantar fasciitis, medial tibial stress syndrome, and stress fractures [8, 9]. Finally, confounding factors such as participants' training history, pre-existing gait adaptations, or genetic predispositions [30] were not accounted for, which may influence injury susceptibility. Further investigations into footwear modifications or orthotic interventions could help refine biomechanical strategies for injury prevention among HA runners.

## 5. Conclusions

This study reveals that acute HI ergometer exercise significantly alters plantar biomechanics in young HA runners, shifting loading toward the medial foot and increasing FPA. These changes may heighten the risks of injuries like plantar fasciitis, medial tibial stress syndrome, and ACL tears [53–55]. For practical application, coaches and athletes should consider periodic biomechanical assessments to monitor runners' responses to HI training, particularly in HA individuals prone to altered loading patterns [9, 47]. Periodized training regimens with adequate recovery intervals may mitigate overload. Training interventions should focus on progressive load management, incorporating lower-limb strengthening exercises, particularly for the medial foot musculature to counteract excessive FPA deviations. Additionally, postexercise gait retraining to correct FPA deviations and targeted neuromuscular exercises, potentially reducing knee joint strain [15, 51]. Real-time biofeedback tools could aid in optimizing foot strike patterns. Adopt preventive interventions, such as custom orthotics to redistribute plantar pressures [18] or strength exercises targeting intrinsic foot muscles and the medial longitudinal arch [7, 11]. Clinicians might also recommend individualized orthotic solutions to provide additional medial foot support [22, 55] and mitigate excessive biomechanical stress.

Future research should explore interactions between footwear design (e.g., minimalist vs. motion-control shoes), exercise modalities (treadmill vs. outdoor running [20]), and biomechanical adaptations across diverse populations. Such insights will refine evidence-based strategies for injury prevention in athletes.

## Figures and Tables

**Table 1:** The participants' physical fitness information.

Variables	Values
One leg standing (maximal 60 s)	60 s
Number of full stands and sits in 30 s with arms folded across chest	32.1 ± 2.4
Number of right arm curls in 30 s holding a 5 kg dumbbell	25.7 ± 1.2
Chair sit, with leg extended and the hand reaching to the toes, distance from the extended fingers to the tip of the toe (cm)	18.7 ± 6.9
Back scratch, distance between extended middle fingers (cm)	7.6 ± 4.4
Rise from seated position, walk 2.45 m, turn, and return to seat (s)	6.8 ± 0.3
Walk speed in 30 m (m/s)	1.3 ± 0.1
Walk speed in 6 min (m/s)	0.9 ± 0.1

*Note:* The data in the table is represented by mean ± SD, *n* = 18.

**Table 2:** The participants' HR and BP pre- and postexercise.

Variables	Values
HR at rest	65.4 ± 6.5 BPM
Estimated HR_max_	198 ± 0.5 BPM
HR reserve (HRR)	132.6 ± 6.6 BPM
SBP at rest	119.8 ± 7.4 mmHg
DBP at rest	65.6 ± 5.6 mmHg
HR_mean_ during exercise	160.6 ± 10.8 BPM
HR_max_ during exercise	178.4 ± 6.9 BPM
HR at 2 min postexercise	124.9 ± 8.0 BPM
SBP at 10 min postexercise	113.7 ± 4.8 mmHg*⁣*^*∗∗∗*^
DBP at 10 min postexercise	60.8 ± 4.5 mmHg*⁣*^*∗∗∗*^

*Note:* The data in the table is represented by mean ± SD, *n* = 18.

*⁣* ^*∗∗∗*^ *p* < 0.001 vs. SBP at rest or DBP at rest.

## Data Availability

The datasets of this study are available from the corresponding author upon reasonable request.

## Nomenclature

ACL: Anterior cruciate ligament
AHI: Arch height index
BMI: Body mass index
BMR: Basal metabolic rate
BP: Blood pressure
BPM: Beats per minute
DBP: Diastolic blood pressure
FI: Fatigue index
FPA: Foot progression angle
FPI: Foot posture index
FTI: Force–time integral
GRF: Ground reaction force
HR: Heart rate
HI: High-intensity
HR_max_: Maximum heart rate
HR_mean_: Mean heart rate
RPE: Ratings of perceived exertion
SBP: Systolic blood pressure
VGRF: Vertical ground reaction force.

## Supporting Information

Supporting Information The fundamental characteristics of the study participants are presented in Table S1. Table S2 details the RPE scores obtained from the participants immediately following the completion of the ergometer exercise.
