# Supplementary material for: Plantar Biomechanic Characteristics After High-Intensity Exercise in Young Runners With High-Arched Feet
Source: Appl Bionics Biomech. 2025 May 20;2025:7834542. doi: 10.1155/abb/7834542 (PMC12116207; doi:10.1155/abb/7834542)
Supplement: Supporting Information — The fundamental characteristics of the study participants are presented in Table S1. Table S2 details the RPE scores obtained from the participants immediately following the completion of the ergometer exercise. [file 7834542.f1.docx]

**ONLINE RESOURCES (supplementary materials)**

**Plantar Biomechanic Characteristics after High-Intensity Exercise in Young Runners with High-Arched Feet**

**Xindong Tao, ^#^ Weiyi Lao, ^#^ Yaoyao Zhong, ^#^ Jihui Wang, and Wei Ouyang***

*College of Physical Education and Health Sciences, Zhejiang Normal University, Jinhua 321004, China*

Correspondence should be addressed to Wei Ouyang; [wouyang@zjnu.cn](mailto:wouyang@zjnu.cn) (W. Ouyang)

^#^These authors contributed equally to this work.

Supplementary Table S1 The participants’ basic data

| Participant | Age  (yr) | Heght (cm) | Weight (kg) | BHR (BPM) | SBP (mmHg) | DBP (mmHg) | Overall fat % | Trunk fat % | LULF % | RULF % | LLLF % | RLLF % | BMI | BMR (KJ) |
| --- | --- | --- | --- | --- | --- | --- | --- | --- | --- | --- | --- | --- | --- | --- |
| #1 | 20 | 175 | 67 | 65 | 125 | 70 | 12.3 | 13.1 | 10.4 | 10 | 12.1 | 11.6 | 21.9 | 7088 |
| #2 | 19 | 182 | 70 | 62 | 127 | 66 | 10.9 | 10.4 | 8.3 | 6.9 | 12.2 | 12.5 | 21.1 | 7653 |
| #3 | 19 | 178 | 62 | 68 | 111 | 60 | 8.0 | 6.5 | 6.1 | 6.3 | 10.8 | 10.2 | 19.6 | 6841 |
| #4 | 20 | 183 | 70 | 62 | 118 | 62 | 18.8 | 20.8 | 13.6 | 14.1 | 17.0 | 16.9 | 21.0 | 6937 |
| #5 | 19 | 181 | 74 | 75 | 133 | 78 | 16.3 | 16.7 | 12.1 | 11.3 | 16.7 | 17.0 | 22.6 | 7527 |
| #6 | 20 | 176 | 74 | 55 | 127 | 68 | 13.4 | 12.5 | 11.3 | 10.6 | 14.9 | 15.4 | 23.9 | 7803 |
| #7 | 20 | 180 | 65 | 76 | 130 | 72 | 12.2 | 13.0 | 9.8 | 9.2 | 11.6 | 11.8 | 20.1 | 6983 |
| #8 | 20 | 179 | 64 | 58 | 118 | 65 | 12.4 | 12.9 | 9.8 | 11.0 | 12.2 | 12.1 | 20 | 6740 |
| #9 | 19 | 179 | 62 | 66 | 128 | 70 | 9.8 | 8.8 | 6.2 | 6.3 | 12.3 | 12.1 | 19.4 | 6724 |
| #10 | 20 | 181 | 68 | 65 | 128 | 80 | 10.0 | 8.6 | 8.5 | 7.5 | 11.8 | 12.9 | 20.8 | 7422 |
| #11 | 20 | 182 | 75 | 57 | 126 | 70 | 8.3 | 4.7 | 18.5 | 2.6 | 17.0 | 11.0 | 22.6 | 7893 |
| #12 | 21 | 181 | 75 | 80 | 112 | 62 | 19.8 | 20.9 | 13.7 | 12.3 | 20.1 | 19.8 | 22.9 | 7330 |
| #13 | 20 | 179 | 72 | 59 | 114 | 61 | 15.3 | 15.8 | 11.4 | 10.8 | 15.6 | 15.9 | 22.5 | 7368 |
| #14 | 20 | 188 | 72 | 64 | 122 | 56 | 18.5 | 16.2 | 12.7 | 10.9 | 17.8 | 16.7 | 20.4 | 8079 |
| #15 | 20 | 179 | 68 | 70 | 101 | 52 | 11.3 | 11.8 | 9.9 | 8.7 | 11.9 | 11.7 | 21.2 | 7068 |
| #16 | 20 | 180 | 78 | 62 | 124 | 60 | 18.4 | 17.6 | 11.8 | 11.2 | 16.6 | 15.8 | 24.1 | 7182 |
| #17 | 21 | 175 | 71 | 68 | 113 | 68 | 13.8 | 14.8 | 10.9 | 10.3 | 13.8 | 13.1 | 23.2 | 7235 |
| #18 | 21 | 172 | 61 | 66 | 114 | 61 | 13.5 | 12.7 | 10.9 | 10.2 | 14.8 | 14.3 | 20.6 | 6895 |

n=18. BHR, basal heart rate; BPM, beat per minute; SBP, systolic blood pressure; DBP, diastolic blood pressure; LULF, left upper limb fat; RULF, right upper limb fat; LLLF, left lower limb fat; RLLF, left lower limb fat; BMI, body mass index; BMR, basal metabolic rate

Supplementary Table S 2 The participants’ rating of perceived exertion after 5 min high--intensity ergometer cycling

| Grade | Rating of Perceived Exertion | RPE n |
| --- | --- | --- |
|  |  |  |
| 6 | No exertion at all |  |
| 7 | Extremely light |  |
| 8 |  |  |
| 9 | Very light |  |
| 10 |  |  |
| 11 | Light |  |
| 12 | Somewhat hard |  |
| 13 |  |  |
| 14 |  |  |
| 15 | Hard(heavy) | 2 |
| 16 | Very hard | 2 |
| 17 |  | 8 |
| 18 |  | 1 |
| 19 | Extremely hard | 5 |
| 20 | Maximal exertion |  |
